# Can Technology Use Alleviate Workforce Needs in Finnish Assisted Living Services? A Convergent Mixed‐Methods Study of Automatic Medicine Dispensers and Night‐Time Monitoring

**DOI:** 10.1155/jonm/2309539

**Published:** 2026-05-08

**Authors:** Visa Väisänen, Henrika Karhulahti-Nordström, Petra Saukkonen, Salla Ruotsalainen, Kim Josefsson, Timo Sinervo

**Affiliations:** ^1^ Health and Social Service System Research Team, Welfare State Research Unit, Finnish Institute for Health and Welfare, Helsinki, Finland, thl.fi; ^2^ Department of Health and Social Management, Faculty of Social Sciences and Business Studies, University of Eastern Finland, Kuopio, Finland, uef.fi; ^3^ Older People Services Team, Services Unit, Finnish Institute for Health and Welfare, Helsinki, Finland, thl.fi

**Keywords:** assisted living, medicine dispensing, night-time monitoring, nursing, technology use, workforce needs

## Abstract

**Background:**

Workforce needs are growing in older people care, exacerbated by the aging population. Technological solutions offer potential means to address this challenge. However, their effects on work and worktime management remain unclear, especially in the setting of assisted living services. Our aim was to assess the potential of two established technologies, automatic medicine dispensing and night‐time monitoring, in alleviating workforce needs in Finnish assisted living facilities.

**Methods:**

The effects of the two technologies on workforce needs were studied using a convergent mixed‐methods design, incorporating quantitative time and motion analysis and qualitative analysis of two open‐ended survey questions. Linear regression analysis and data‐driven thematic analysis were employed. The quantitative and qualitative results were integrated and compared afterward.

**Results:**

Our quantitative results indicated that medicine dispensing technology can streamline medicine management and administration, but effects on direct care time were not found. Use of night‐time monitoring technology was associated with more direct care during nights and reduced nightly staffing. The qualitative analysis highlighted medicine dispensers freeing up time for other work and night‐time monitoring reducing the need for nightly rounds. However, several technology‐related problems and a loss of medication competencies were perceived. Our results converged on several positive workforce effects, notably optimizing medication management and promoting individualized care during nights.

**Conclusion:**

Technology use could be promoted in assisted living services, when it demonstrably streamlines care work, increases work efficiency, allows for reduced staffing, or promotes individualized and patient‐centered care. National care policy can incentivize the use of similar technologies, for example, through staffing level legislation. However, care unit management should prepare for potential technology‐related errors and acute emergencies by upkeeping both technology and medication competencies of nurses and ensuring the presence of sufficient staff. Importantly, the potential efficiency gains must not come at the expense of quality or safety of care.

## 1. Introduction

Services for older people across the world are challenged by aging populations [1] and dwindling workforces [2]. To meet the growing care demands of current older people and those soon entering the formal care systems, service provision must advance especially in its effectiveness. This means that more must be done with fewer resources. Among developments targeting the workforce, such as increased involvement of informal carers [3] and international recruitment of nurses [4], rapidly advancing technologies could also alleviate the workload of nurses, freeing up time for more direct care with clients, and in this way influence the demand for workforce [5, 6]. Compared to assisted living facilities, the use of technology in home care services is more established. For instance, technologies related to information and communication [7], medicine dispensers [8], smart home solutions [9], and enterprise resource planning (ERP) systems [10] have been extensively researched and their use in practice is frequent. In contrast, the use of technology in assisted living facilities is not as commonplace. While manual alarming systems [11], some transfer aids [12], and information and communications technology solutions [13] are used, transformative care technologies relevant for increasing the efficiency of care work are not widely utilized [14, 15].

Research on the workforce effects of technology used in assisted living has focused on monitoring, robotics, medication management or administration, and ERP systems. However, evidence on the topic is scarce, and the results found pertain to single studies. Research on monitoring technologies consists of module‐based automatic monitoring and documentation system [16] and AI‐based monitoring, which identified changes in health status [17]. These technologies enabled faster responsiveness of care and freed up workforce resources, regarding both the number of employees needed and the time needed to complete tasks. Research on robotics concerns transfer aids, which have managed to reduce the workforce resources needed to transfer clients [18] and various other mobility and communication robots, which have reduced the labor costs of the organizations, while promoting flexible work arrangements [19]. Evidence on medication technologies include computerized ordering systems and medication administration records, which have had mixed results on workforce needs [20] and marginal reductions in the time spent on medication management or administration [21]. Lastly, varying ERP systems, some based on cloud‐based solutions, have increased the efficiency of work by optimizing the use of workforce resources [22–24] by, for instance, accounting for the number of employees and the client’s treatment plans.

In this study, we examine two established technologies in Finnish assisted living services, which were night‐time monitoring technology and automatic medicine dispensing (mainly referring to individual unit‐dose bags automatically packed by pharmacies). The use of other technologies was marginal or they were not in established use yet. Approximately 41% of the assisted care units utilized night‐time monitoring technologies, which varied somewhat in their implementation, and 84% of units had medicine dispensing technology in use [25]. These technologies can reconfigure work through automation effects, for instance by replacing previous human inputs in medicine management and monitoring of residents during nights. However, they can also augment daily work by enabling less error‐prone, flexible, and timely medication management and administration, as well as better directing nightly care resources to those in need. These previously separate automation and augmentation functions have been recently understood as interlinked, emphasizing the transformative workforce potential of technology use [26, 27]. The main mechanism through which these technologies can affect workforce outcomes is improved task efficiency (e.g., less time spent on medicine management or more needs‐based allocation of limited night‐time resources). These technologies can also directly enable better staffing optimization (i.e., reduction or reconfiguration of staff during night shifts by reducing cognitive or clinical workloads). In addition, through more optimized workflows enabled by technology use, a potential pathway of increased task efficiency leading to reduced staffing needs emerges. This phenomenon is likely to be further accelerated by the emergence of AI‐driven solutions. In the context of a setting facing workforce challenges, efficiency gains may lead to doing the same work but with fewer resources.

Research on medicine dispensing focuses heavily on home care or health care settings [8, 28, 29], with studies conducted in assisted living facilities or nursing homes largely missing. Medicine dispensing systems in acute care hospitals can improve safety and quality of care by decreasing medication errors (excluding prescribing errors), save employee time especially when using centralized systems, and might reduce costs especially in larger units with better care resources [30]. In emergency departments, dispensing machines were found to delay retrieval of certain medications while expediting the retrieval of more controlled medications [31]. However, the staff perceived medication retrieval times as faster, highlighting the importance of not only using quantitative measures when assessing the impact of technologies.

Night‐time monitoring technologies have been examined also in the care setting of older people, and there have been calls for interventions that improve night‐time care for both the residents and the employees [32]. Various sensor technologies researched often target fall risk reduction, supporting or engaging clients with dementia, and independent living promotion [33]. The results of the technology use vary, but moderate reductions in falls and some cost savings have been found [33]. Monitoring systems can also improve residents’ sleep quality through helping nurses plan their night‐time care activities in accordance with clients’ individual needs by, for example, identifying patterns of mobility eventually resulting in customized care plans [34]. Similarly, monitoring technology can reduce the need for night‐time rounds and manual checking, which can promote the sleep of residents and consequently improve their ability to participate in daily activities [35]. While the use of technologies can be especially useful for caring for clients with dementia, special user requirements such as early implementation and gradual familiarizing should be considered [36]. Importantly, research on the direct workforce effects of night‐time monitoring, relevant for alleviating workforce needs, is largely missing.

To summarize, there is a lack of evidence on the workforce effects of both medicine dispensing and night‐time monitoring technologies in assisted living services for older people. Previous research in adjacent care settings suggests that medicine dispensing can lead to some reductions in time spent on medicine‐related tasks, but with some implementation‐related challenges related to sudden changes in medicine regimens. Use of night‐time monitoring technologies has been associated with reduced need for nightly rounds, thus potentially resulting in task optimization. Information about the workforce effects of these technologies is crucial to support decision‐making at both local and national levels, for example, by enabling managers of care units to make informed decisions about technology introduction and implementation. In addition, if these technologies prove beneficial for reducing workforce needs, national care policy can incentivize the deployment and utilization of technologies among care providers.

To fill the identified research gap, we examined the use of two established technologies (automatic medicine dispensing and night‐time monitoring). As previous research has indicated that the views of staff might differ from purely quantitative objective measurements, we employed a convergent mixed‐methods design. This approach allows us to separately collect and analyze quantitative and qualitative data and compare their results to gain a better understanding of the potential effects of technology use on workforce needs. We formulated two research questions:

1. How can the use of automatic medicine dispensing and night‐time monitoring technologies affect workforce needs in assisted living services? (quantitative)
2. How do nurses perceive the use of automatic medicine dispensing and night‐time monitoring technologies in assisted living services? (qualitative)

## 2. Materials and Methods

### 2.1. Design

The study utilized a convergent mixed‐methods study design [37]; quantitative with a cross‐sectional time and motion study, and qualitative with two open‐ended survey questions. Quantitative and qualitative data were collected and analyzed simultaneously but separately. Following the individual analyses, the authors reviewed the results emerging from both qualitative and quantitative components as well as worked together to integrate and compare them. The Good Reporting of A Mixed Methods Study (GRAMMS) guidelines were followed [38].

### 2.2. Study Setting

In Finland, there has been a persistent trend away from institutional care, and the current older people care policy emphasizes aging in place. Care for older people consists mainly of home care, incorporating home nursing and home help services, and long‐term care comprising assisted living services, mainly with 24/7 assistance available. In this study, only facilities with 24 /7 services participated. These services are provided by 21 wellbeing services counties (and the city of Helsinki), which are responsible for health and social services. These counties have populations ranging from 67,995 to 689,768, with significant variation in population density and geographical size. Admission to home care and assisted living services is gated by assessment of care needs. As of 2024, 6.4% (41,972) of older people aged 75 or more are residents in assisted living facilities [39]. While most of the home care providers are public, over half of the assisted living units are privately owned (but most of their funding comes from the public sector). There is staffing ratio legislation in place mandating the number of care staff per client, with the current minimum staffing ratio being 0.60 as of 2025. The regulatory authorities may also require higher staffing levels and a certain number of employees during night shifts, depending on the residents’ care needs. The present government has introduced legislation that allows care units to lower their staffing levels at most by 0.04 to a minimum of 0.60, if care technologies that could replace care work are in use [40].

The present study focuses on assessing in‐use technologies, which do not currently integrate AI. In Finland, various AI‐driven solutions are presently being developed (see work organized by DigiFinland), with a particular focus on automatic nursing documentation. However, during our data collection, these AI‐driven technologies were still in their development or pilot phases and, as such, were omitted from this study. In addition, various adaptive optimization models were not examined, as Finland utilizes a mandated static staffing ratio. Consequently, in the manuscript, we chose to limit our engagement with recent AI‐driven and dynamic approaches to staffing optimization.

### 2.3. Data Collection

The participating units were recruited through direct e‐mails to all service providers, adverts in traditional and social media, and letters distributed through various stakeholders such as labor market organizations. Quantitative data were collected using paper time measurement forms (see supporting information) distributed to the units taking part in the study. The forms included an information sheet about the study as well as directions and an example on how to fill the forms. The time measurement forms and their tasks were adapted from a previously conducted time measurement study [41, 42]. Data collection was conducted in spring of 2024, lasting for one week. In principle, all care professionals working in the units during this week participated in the study alongside their work. Informed consent was assumed upon filling and returning of forms. The forms were returned to the sender (a contracted company), which in accordance with data regulations scanned and digitalized the information into a data file. The data were checked and cleaned, for example, by clarifying unclear markings together with the project researchers. The survey data were merged with unit‐level register data from the resident assessment instrument (RAI) assessments and the monitoring of services for older people (VanPal) survey, both collected by the Finnish Institute for Health and Welfare.

Qualitative data were collected through a personnel survey. The survey was conducted in spring of 2024 in the assisted living units that signed up for the study. A total of 225 employees responded to the survey. The survey, encompassing various work‐related well‐being questions, also included two open‐ended questions about the use of technology in the employees’ work. First, the respondents were asked to describe how medicine dispensing technology had affected their own work and care of residents if the unit used related technology (e.g., pharmacy unit‐dose bag dispensing or medicine robots). This open‐ended question was answered by 89 (40%) employees, with all responses concerning experiences regarding automatic medicine dispensing by pharmacies. In the second question, the respondents were asked how technology related to night‐time monitoring had affected their own work and care of residents if the unit utilized related technology. This question was answered by 101 (45%) employees.

### 2.4. Study Measures

The main measures of interest, whether the unit used automatic medicine dispensing or night‐time monitoring technology, were determined from a survey conducted for the managers of the care units. In the survey, “medicine dispensing” was defined as technologies that assist in medication management or administration (e.g., the distribution or handling of medicines). This included automatic medicine reminders, medicine robots, digital pill dispensers, or automatic medicine dispensing (into ready to distribute unit‐dose bags) done in the pharmacy. Vast majority of the medicine technology used in the care units was automatic medicine dispensing done by a pharmacy, which refers to machine‐packed medications per resident, removing the need for nurses in care units to handle medicines manually (except for distribution). “Night‐time monitoring” on the other hand was defined as technology that help monitor the condition or movement of residents during the night, alerting the employees (often in the office or through a mobile device) in case of unexpected movement (e.g., a fall) or an emergency. This included electronic wearables, intelligent floors or carpets, and motion detectors. Automatic alerting or reporting to the employees was required, and as such, wearables requiring manual operation by the residents were not deemed as monitoring technology.

The outcome measures examined included time spent on direct care time and on medication management or administration, and the number of employees during night shifts. These outcomes varied at the level of the work shift. The outcomes on direct care time and medication management or administration were determined from the forms by counting the total time marked for these tasks. If multiple tasks overlapped, the time was divided evenly between them. The number of employees during shifts was determined by counting the number of shifts (i.e., employees working) during different times as indicated by the forms, and this information was aggregated to the unit level.

Additional adjusting variables, which could potentially bias the association between technology use and the outcomes, were included. These encompassed information about the time of the shift (morning, day/evening, or night), day of the week (weekdays, Saturday, or Sunday), and the occupation of the employee completing the shift (licensed practical nurse, registered nurse, nurse assistant, or other). In a previous time measurement study, worktime spent on direct care time and other tasks, such as medication management, differed depending on the occupation, time of the day, and day of the week, among other factors [41, 42].

In addition, to account for the care needs and the workforce situation of the care units, variables on the staffing level (number of employees per client) and the case‐mix index were included. These variables varied at the level of the care unit. The residents’ mean case‐mix index (RUG‐18) was determined from RAI assessments and correspond to the care needs of the clients, more specifically the cost‐adjusted care time needed compared to the average client (1.0). For instance, a client with an index value of 0.90 requires approximately 10% fewer care resources (i.e., care time) than the average client. Lastly, information on whether the unit was public or private was included. These factors have been previously associated with received care time among residents in Finnish assisted living facilities [41].

### 2.5. Data Analysis

Quantitative analysis was done to examine the statistical associations of technology use and workforce needs. First, we used independent samples *t*‐test to compare the size, employee and resident characteristics, and time spent on direct care time and medication management or administration of the care units by their technology use. Next, linear regression models were built, which examined the association of the use of technology on the outcome variables. The models were adjusted for shift and unit characteristics. Models 1 and 3 investigated the worktime spent on direct care, model 2 investigated the worktime spent on medication management or administration, and model 4 investigated the number of employees working during night shifts.

Models 1–3 were analyzed using work shift–level data (*n* = 1318 in models 1 and 2, and *n* = 395 in model 3), while model 4 included organization‐level data (*n* = 52). To account for the nested data structure at the care unit level (i.e., within‐cluster correlation), the standard errors of models 1–3 were adjusted for clustering. This results in correction of the assumption of independence of observations, providing more accurate confidence intervals and *p* values [43]. Data management and statistical analyses were done with R Statistical Software version 4.2.3 for Windows 11 [44].

Qualitative data were analyzed using inductive thematic analysis [45]. Data were coded separately for night surveillance technology and medicine dispensing technology. In the survey, open‐ended answers were given as individual words or a few sentences. One researcher (H.K‐N.) read through the responses three times to gain a comprehensive understanding of the data. Responses were independently coded by H.K‐N., using the qualitative data analysis software Atlas.ti version 24. A word or a sentence was used as the unit of analysis, depending on the length of the response. Single‐word responses were used directly as codes, and longer responses were abbreviated into codes that closely reflected the original expressions. Codes were organized into groups based on content similarity and exported to Excel for further structuring. The coding was used to form themes that described the respondents’ experiences.

The codes were combined into subcategories (medicine dispensing *n* = 4, and night‐time monitoring *n* = 6), which were combined into two upper categories in each topic (benefits and challenges). To ensure credibility, the encodings and classifications were reviewed together with two other researchers (T.S. and V.V.), with H.K‐N. maintaining the documentation of the process. The project group also reviewed the results to ensure consistency and relevance to the research questions, which supported the reliability of the research. In addition, direct quotes from the answers were used in the results (the answers were translated from Finnish to English).

Finally, the main results of the quantitative (i.e., estimates) and qualitative analyses (i.e., themes) were integrated. We analyzed the convergence and divergence by utilizing side‐by‐side mapping of found results. For instance, if the quantitative analysis indicated a specific result, we examined whether this finding (considering both the direction and magnitude) emerged in the qualitative analysis, and vice versa. This integration enabled us to expand our understanding of both the quantitative and qualitative results, while strengthening the validity of the study [46].

### 2.6. Ethical Considerations

The Ethical Review Board of the Finnish Institute for Health and Welfare approved the study (THL/1245/6.02.01/2024). Individual research permits were applied for if the care organization required them. Study participants were informed about the study through an information sheet. Participation in the study was voluntary, and anonymity of the participants was guaranteed. Filling and returning the forms were seen to indicate informed consent to participate. The study adhered to the ethical principles of Declaration of Helsinki.

## 3. Results

### 3.1. Quantitative Results

The units using the established technologies of medicine dispensing and night‐time technology differed somewhat from units not using these technologies (Tables 1 and 2). However, most of the differences were not statistically significant. For medicine dispensing, the units utilizing the technology had residents with lower care needs (3.24 vs. 3.47, *p* = 0.066) and used marginally less time on medication management or administration (2.7% vs. 3.3%, *p* = 0.136) than units not utilizing the technology. Units using night‐time monitoring technology were more often private organizations (61.7% vs. 33.3%, *p* = 0.053), had lower care‐need residents (3.26 vs. 3.50, *p* = 0.069), and had fewer staff during night shifts (0.043 vs. 0.063, *p* = 0.023) than units not utilizing the technology.

**TABLE 1:** Comparison of care unit characteristics by medicine dispenser technology use (*n* = 55).

	Medicine dispenser technology (*n* = 55)			
	Yes (*n* = 30)	No (*n* = 25)	Independent samples *t*‐test	
	Mean (SD)	Mean (SD)	*t*‐statistic	*p* value
Unit size				
Residents	22.7 (10.8)	24.8 (10.1)	−0.73	0.471
Employees	18.3 (10.8)	18.8 (9.2)	−0.20	0.841
Forms returned (i.e., number of work shifts)	64.1 (37.2)	75.0 (38.3)	−1.06	0.294
Unit characteristics				
Private organization	56.7%	48.0%	0.63	0.531
Case‐mix index	0.964 (0.034)	0.977 (0.037)	−1.36	0.180
ADL of residents (1–5)	3.24 (0.43)	3.47 (0.44)	−1.87	0.066
Staffing ratio	0.686 (0.037)	0.696 (0.060)	−0.72	0.475
Worktime (%) spent on				
Direct care	49.7% (5.3)	51.2% (6.4)	−0.91	0.366
Medication management or administration	2.7% (1.0)	3.3% (1.7)	−1.52	0.136

*Note:* Most units (excluding three) were part of both the medicine dispenser and night‐time monitoring groups. The total number of care units in the study was 55.

**TABLE 2:** Comparison of care unit characteristics by night‐time monitoring technology use (*n* = 52).

	Night‐time monitoring technology (*n* = 52)			
	Yes (*n* = 34)	No (*n* = 18)	Independent samples *t*‐test	
	Mean (SD)	Mean (SD)	*t*‐statistic	*p* value
Unit size				
Residents	23.5 (9.0)	22.7 (11.8)	0.27	0.788
Employees	19.1 (10.2)	17.1 (10.0)	0.68	0.498
Forms returned (i.e., number of work shifts)	64.8 (38.5)	77.2 (37.4)	−1.13	0.266
Unit characteristics				
Private organization	61.7%	33.3%	2.00	0.053
Case‐mix index	0.966 (0.039)	0.978 (0.033)	−1.23	0.227
ADL of residents (1–5)	3.26 (0.47)	3.50 (0.38)	−1.87	0.069
Staffing ratio	0.683 (0.046)	0.705 (0.054)	−1.48	0.149
Nightly staff per resident	0.043 (0.028)	0.063 (0.030)	−2.38	0.023
Worktime (%) spent on				
Direct care	50.0% (6.0)	51.1% (5.9)	−0.61	0.546

*Note:* Most units (excluding three) were part of both the medicine dispenser and night‐time monitoring groups. The total number of care units in the study was 55.

The results of the fully adjusted linear regression analysis are presented in Table 3. The use of medicine dispensing technology in the unit was not associated with increased direct care time given (*β* = 4.79, 95% CI: −16.02–25.60). However, work shifts done in units with medicine dispensing technology used approximately 10 minutes less time on medication management or administration (*β* = −10.45, 95% CI: −16.58–4.32). This equates with an approximate 2% saving in worktime, when assuming an average shift length of 8 h.

**TABLE 3:** Results of the linear regression analysis examining the association between technology use and worktime or staffing.

	Model 1: Direct individual care time (minutes)	Model 2: Medication management or administration (minutes)
	β (95% CI)	β (95% CI)
Medicine dispensing technology in use		
Yes	4.79 (−16.02–25.60)	−10.45 (−16.58 to −4.32)^**∗∗∗**^
No (ref.)		
Adjusted *R* ^2^	0.043	0.054

	**Model 3: Direct individual care time (minutes)**	**Model 4: Number of care personnel per resident during night shifts**

Night‐time monitoring technology in use		
Yes	45.38 (9.75–81.00)^**∗**^	−0.02 (−0.04 to −0.00)^**∗**^
No (ref.)		
Adjusted *R* ^2^	0.105	0.124

*Note:* The models were adjusted for work shift, day of the week, occupation, care organization type, and unit characteristics. See supporting file for full results. The models 1, 2, and 3 were adjusted for clustered standard errors on the level of the organization. Models 1 and 2 included only shifts with medication management time (*n* = 1318). Model 3 only included night‐time shifts (*n* = 395). The models were adjusted for controlling variables.

^∗^ *p* < 0.05.

^∗∗^ *p* < 0.01.

^∗∗∗^ *p* < 0.001.

Use of night‐time monitoring technology was associated with 45 minutes of increased direct (individual) care time per night shift (*β* = 45.38, 95% CI: 9.75–81.00). In addition, on an organizational level, care units utilizing night‐time monitoring technology had fewer care personnel during night shifts (*β* = −0.02, 95% CI: −0.04 to 22120.00). This estimate corresponds to a staffing reduction equivalent to half of a full‐time care personnel per night shift in a typical care unit with 24 residents.

### 3.2. Qualitative Results

#### 3.2.1. Perceptions of Medicine Dispensing

The respondents experienced both benefits and challenges on pharmacy prepared medication unit‐doses. The themes that emerged were focused on time management and medication safety. Of the 89 responses, 48 identified that this technology allowed them more time with residents and the opportunity to complete other unit tasks while 18 stated that it increased their medication management workload. Medication safety was identified as improved by 11 respondents while some expressed concerns that it decreased medication safety (*n* = 5).

Medicine dispensing was perceived to free up more time for client work and other tasks by clarifying and speeding up the work related to medication management or administration. The dose bags contain all the essential information related to the administration of the resident’s medication, which was seen to both facilitate the distribution of medicines and reduce the possibility of medicine errors.

“Time is freed up for residents, as previously time was spent on distributing medicines.”

“I find that the medicine dispensing makes medication management much easier and clearer.”

However, some respondents indicated that medicine dispensing technology required greater care in checking and administering medicines. Making changes to the medication regimens was also perceived as stressful. Notifying the pharmacy of the changes and adding or removing medicines from the bags was considered time‐consuming.

The respondents highlighted that the technology use led to distancing themselves from the residents’ medication. This was seen to have a negative impact on the nurses’ competence in medication management and their comprehensive understanding of the residents’ medicine needs.

“Medicine dispensing slows down work and you lose your skills in identifying medicines.”

“[The technology] speeds up the process of dispensing medicines, but the identification of medicines gets worse over time.”

#### 3.2.2. Perceptions of Night‐Time Monitoring Technology

Similarly, the respondents (*n* = 101) experienced both benefits and challenges with night‐time monitoring technology.

With the help of technology, employees received information about possible movements or need for help without having to visit residents’ rooms unnecessarily. This was perceived to be useful, as it enabled more efficient allocation of work tasks during nights and reduced the need to wake up residents. Employees noticed that residents slept better when the regular night‐time rounds were reduced. In addition, the technology also made nurses feel safer by increasing their awareness regarding the status and activity of the residents.

“Technological solutions have made the residents’ sleep much calmer, as the nurses do not have to visit the rooms every now and then to check that the residents have not fallen, for example.”

“You know more quickly who needs help at any given time. Providing care is faster. I also feel safer knowing when a resident is on the move.”

Employees were particularly burdened by technical problems, such as delayed arrival of alarms or erroneous alarms caused by the device’s oversensitivity to movement. In addition, some employees felt that they and the residents did not have enough expertise to make effective use of the technology.

“Technological devices can be helpful, but they are not used properly.”

“If the [night‐time monitoring] technology worked well, it would help a lot in the workplace. But it doesn’t.”

## 4. Discussion

This research provided insights and addressed a gap in the literature on two technologies, medicine dispensing (e.g., automatic unit‐dose bag packing done by pharmacies) and night‐time monitoring, that can potentially reduce workforce needs in assisted living services. Our results indicate that medicine dispensing technology can reduce work time spent on medication management or administration, but no effects on the amount of direct care time were observed. The qualitative analysis highlighted similar benefits; however, the respondents also indicated that more time was made available for residents, a result not supported by our quantitative results. In addition, the technology was seen to enhance the safety of residents and reduce medicine errors. However, potential challenges, such as changes in medication regimes and competency loss, were also identified. Results on night‐time monitoring technology’s effects converged on the potential increase in nightly individual direct care time, but allowing for reduced care personnel during the nights only emerged in the quantitative analysis. Nurses indicated that nightly check‐ups were not needed; instead, automatic alarming enabled more efficient allocation of nightly work time. In addition, safety of both the residents (faster reaction to emergencies and falls) and the employees (heightened situational awareness) was brought up by the respondents. However, some perceived the technology as largely nonfunctional or poorly configured, and that it was not used properly due to the employees’ lacking technological competence. To summarize, our integrated findings converged on positive workforce effects of the examined technologies, with some divergence on the specifics (e.g., benefits on direct care time and reduced nightly staffing).

For managers of assisted living facilities, the technologies examined in this study can offer opportunities to further optimize the use of workforce resources and increase efficiency. This was especially apparent for night‐time monitoring technology, which could at least in larger care units allow for significantly reduced staffing during night shifts. Similarly, streamlining the work related to medication management or administration can help management address workforce demands by freeing up time that can be spent on other work tasks. However, the introduction of technologies might not automatically lead to work time savings. In the present study, the technologies were already in established use, with assumed successful implementation. This often includes training the nurses to use the technology, modifying workflows to accommodate the use of technology, developing management practices to facilitate the use of technology, and maintaining the required competences related to it. Promoting the digital capability of nurses [47] as well as clearly communicating the practical and clinical benefits of technologies [48] emerge as key factors of successful technology implementation. Moreover, our study was conducted in the context of the Finnish health and social care system, where the staffing levels are regulated and the care units are periodically monitored. Consequently, accounting for the local and organizational contexts remains a necessary step when planning, implementing, or using care technologies [48].

This study provided evidence that medicine‐dispensing and night‐time monitoring technologies can positively affect workforce needs in assisted living facilities setting. Our results concerning medicine dispensing partly align with previous studies in other care settings, which have associated the use of some medicine dispensing technologies with reduced workloads and costs [28, 30]. However, the potential effects might be limited if residents of the unit require frequent medication changes (i.e., instabilities in health, see for example [49]). Further research is warranted, particularly regarding pharmacy‐packed unit dose bags in long‐term care settings. While night‐time monitoring systems have also been scarcely researched, some studies have linked its use to reduced night‐time checking [35], aligning with both our quantitative and qualitative analysis. In addition, marginal cost reductions have been found, potentially pointing to opportunities to reduce nightly staffing levels [33]. If the use of night‐time monitoring technology enables an additional 45 minutes of direct care per night shift, individual care needs of residents can be better met. This better allocation of work time might not only allow for reduced staffing but also permit paying attention to care tasks beyond those strictly necessary, possibly promoting client‐centered and holistic care. It is important to note that potential staffing reductions enabled by night‐time monitoring technologies cannot materialize in smaller care units, which might have only few nightly staff to begin with. Moreover, the safety of residents requires a certain number of employees, which cannot be decreased regardless of technology use. For instance, if a fall occurs, enough nurses must be present to help.

For the use of technology to produce added value in nursing work, it is important to pay special attention to the development of the personnel’s technological competence and the integration of technologies into daily work. The availability of technical support in the event of disruptions is crucial [47], for both the residents and the employees. In addition to digital competencies, maintaining the employees’ expertise in medication management and administration was perceived as important in our results. According to previous research, the medicine‐related competencies of nurses are wide‐ranging, and this plays a key role in promoting quality of care [50]. As such, it is important to reach a balance in streamlining medication management (i.e., freeing up workforce resources for other care tasks) and maintaining professional competencies in case of disruptions or acute circumstances. Some challenges with the use of night‐time monitoring technology also emerged in our qualitative analysis, for instance, the improper use of technology and the technologies not functioning correctly. These aspects can be at least partly addressed by effective adoption and implementation of technologies [51], in addition to working together with vendors to reduce errors and false positives. Lastly, depending on national laws and regulations, several ethical and practical concerns might arise with (night‐time) monitoring [52]. For instance, the residents of Finnish assisted living facilities are living in their homes (rented from the care unit), and consequently written informed consent is often required for various technologies, possibly complicating the introduction of monitoring technologies, particularly for residents with dementia [53].

Our study provides several practical implications, both for general older people care policy and for management of long‐term care providers. As staffing level legislation is in place in Finland, and the use of approved technologies will allow for reduced staffing levels, the importance of continuous evaluation of current and emerging technologies is emphasized. In addition, regardless of technology use, the presence of sufficient staff to respond to acute situations or emergencies is imperative. However, promoting the use of technologies with sufficient evidence of workforce alleviating effects is key, especially in aging societies with limited care workforce capabilities. Emerging AI‐driven solutions in nursing staff allocation and workforce modelling [54, 55] can potentially shift the staffing optimization from static staffing ratios (current approach in Finland) to adaptive and evolving systems, which has implications for staffing‐level legislation. By locating the “sweet spot” between quality of care and costs (i.e., staffing) in real time, aided by adaptive AI‐supported approaches, resiliency and responsiveness of the overall care system are promoted [56, 57]. Future research examining the development and implementation of AI in nursing must consider workforce effects thoroughly, including the preservation of clinical expertise and critical decision‐making, as well as maintenance of human‐centric principles [54, 58]. These considerations remain relevant for the technologies examined in the present study, as future AI integration in medication management and monitoring, as well as in other care work domains, is likely.

In the future, it is crucial to thoroughly review and research the potential effects of new technologies, particularly if they become part of national legislation (and thus their use is highly promoted). The presence of meaningful incentives for the service providers to develop and invest in technologies is also critical. On the care unit level, even without direct incentives provided by staffing‐level legislation, the technologies examined can provide concrete benefits that enhance and streamline the daily work of nurses. In the long run, this can help with local workforce shortages and provide better care for the residents. However, it is critical that the benefits (e.g., alleviating workforce challenges, increasing the efficiency of work) outweigh the potential disadvantages (e.g., reduced staffing levels, detrimental effects on quality or patient‐centeredness of care), in addition to those presented in our qualitative analysis. Lastly, it is necessary to note that our results on the workforce effects contain uncertainties, and the found effects may vary depending on, for instance, the size of the care unit, the care needs of residents, or whether the unit is public or private (which can affect reporting obligations and monitoring).

## 5. Limitations

Our study had some limitations. First, our study samples (i.e., units utilizing technologies) were not randomized, which weakens the level of evidence provided. However, the mixed‐methods design utilized provided more in‐depth information and congruent findings across the quantitative and qualitative methods, strengthening our results. As staff perceptions of the benefits of technology might not always fully align with objective indicators (e.g., worktime allocation), the need for both quantitative and qualitative perspectives is highlighted. Next, the characteristics of units were compared and found to be similar, but some other unadjusted factors could partly explain the differences found. For example, units with technologies in established use might have had better resources for developing their services or employees and management more open to new ways of working. We accounted for client characteristics, the staffing situation, and unit size in our analysis, but other confounding factors might have existed, potentially affecting the estimates. Furthermore, the activities of care units (particularly private units) are often constricted by regulations, monitoring, and contracts, which might limit the generalizability of our results. This could potentially bias our estimates downward, as the observed variance between care units could be limited by these factors. As such, more research is needed to confirm our findings. In the future, randomized designs would be preferred to minimize the potential of bias and confounding.

Next, we did not quantitatively examine the negatives of technology use, such as challenges with implementation, potential malfunctions, or time spent troubleshooting. In addition, we did not examine direct effects on residents, but our qualitative results supplemented the analysis providing insights on perceived benefits and challenges. While technology use might be beneficial for employees and the overall staffing situation in the units, the effects could potentially be different for the residents, for instance by reducing the number of available care staff in case of emergencies or by directly or indirectly reducing time spent on social activities. However, an increase in direct care time during night‐time could potentially enable more individualized care, thereby promoting positive outcomes also for residents. Furthermore, at least in theory, less worktime spent on medication management and administration could increase the time for direct care, but our results did not entirely support this. Consequently, the effects of technology use for residents should be examined in the future.

## 6. Conclusions

We examined the effects of two established technologies (automatic medicine dispensing and night‐time monitoring) on workforce needs in Finnish assisted living facilities. Our mixed‐method analysis approach provided evidence of positive workforce effects, for example, streamlining the work related to medication management or administration, increasing the amount of individualized care during nights, and allowing for reduced nightly staffing. In addition, several perceived challenges with technology use emerged, such as loss of medication competency and technical problems. Our results imply that managers of care units should examine whether these technologies could provide tangible benefits that can alleviate workforce concerns, increase the efficiency of care, and potentially promote more patient‐centered care. National care policy could promote these and similar technologies, which have sufficient evidence of positive effects, in older people care, especially in aging societies with forecasted consequential care workforce shortages. However, the quality and safety of care must be maintained, for instance by ensuring sufficient (nightly) staffing, upkeeping medication competencies, and preparing for potential disruptions or errors with technology use.

**Nomenclature**
ERP: Enterprise Resource Planning system
RAI: Resident Assessment Instrument

## Author Contributions

Visa Väisänen: conceptualization, writing–original draft, methodology, and formal analysis. Henrika Karhulahti‐Nordström: writing–original draft and formal analysis. Petra Saukkonen, Salla Ruotsalainen, and Kim Josefsson: writing–review and editing. Timo Sinervo: writing–review and editing, supervision, and project administration.

## Funding

This research was co‐funded by the Finnish Ministry of Health and the Finnish Work Environment Fund (230337). Open access publishing was facilitated by Terveyden ja Hyvinvoinnin Laitos, as part of the Wiley–FinELib agreement.

## Disclosure

The funders had no role in the writing, editing, approval, or decision to submit this research. All the authors read and accepted the final manuscript.

## Ethics Statement

The Ethical Review Board of the Finnish Institute for Health and Welfare approved the study (THL/1245/6.02.01/2024). Individual research permits were applied for if the care organization required them. Study participants were informed about the study through an information sheet. Participation in the study was voluntary, and anonymity of the participants was guaranteed. Filling and returning the forms were treated as an indication of informed consent to participate. The study adhered to the ethical principles of the Declaration of Helsinki.

## Consent

Please see Ethics Statement.

## Conflicts of Interest

The authors declare no conflicts of interest.

## Supporting Information

Additional supporting information can be found online in the Supporting Information section.

## Supporting information


**Supporting Information 1** Time measurement form used (translated to English).

**Supporting Information 2** Full linear regression results.

## Data Availability

The data that support the findings of this study are available from the corresponding author upon reasonable request.

## References

[1] Rechel B. , Grundy E. , Robine J.-M. et al., Ageing in the European Union, Lancet. (2013) 381, no. 9874, 1312–1322, 10.1016/S0140-6736(12)62087-X, 2-s2.0-84876194638. 23541057

[2] OECD , Who Cares? Attracting and Retaining Elderly Care Workers, 2020, OECD, 10.1787/92c0ef68-en.

[3] Broese Van Groenou M. I. and De Boer A. , Providing Informal Care in a Changing Society, European Journal of Ageing. (2016) 13, no. 3, 271–279, 10.1007/s10433-016-0370-7, 2-s2.0-84963727521. 27610055 PMC4992501

[4] Kingma M. , Nurses on the Move: A Global Overview, Health Services Research. (2007) 42, no. 3p2, 1281–1298, 10.1111/j.1475-6773.2007.00711.x, 2-s2.0-34247884381. 17489915 PMC1955376

[5] Hussain A. , Rivers P. A. , Glover S. H. , and Fottler M. D. , Strategies for Dealing With Future Shortages in the Nursing Workforce: A Review, Health Services Management Research. (2012) 25, no. 1, 41–47, 10.1258/hsmr.2011.011015, 2-s2.0-84857020428. 22323671

[6] Krick T. , Huter K. , Domhoff D. , Schmidt A. , Rothgang H. , and Wolf-Ostermann K. , Digital Technology and Nursing Care: A Scoping Review on Acceptance, Effectiveness and Efficiency Studies of Informal and Formal Care Technologies, BMC Health Services Research. (2019) 19, no. 1, 10.1186/s12913-019-4238-3, 2-s2.0-85067543821. PMC6585079 31221133

[7] Lindberg B. , Nilsson C. , Zotterman D. , Söderberg S. , and Skär L. , Using Information and Communication Technology in Home Care for Communication Between Patients, Family Members, and Healthcare Professionals: A Systematic Review, International Journal of Telemedicine and Applications. (2013) 2013, 1–31, 10.1155/2013/461829, 2-s2.0-84877311623. PMC3649237 23690763

[8] Salmensuu O. , Hyttinen-Huotari V. , Isotalo J. , Rijken M. , Linnosmaa I. , and Kaarakainen M. , Systematic Review of Effects of Medication Dispenser Use by Home-Dwelling Older Adults, Nursing Research. (2025) 74, no. 4, 305–312, 10.1097/NNR.0000000000000824. 40203839 PMC12188797

[9] Martin S. , Kelly G. , Kernohan W. G. , McCreight B. , and Nugent C. , Smart Home Technologies for Health and Social Care Support, Cochrane Database of Systematic Reviews. (2008) 2010, no. 1, 10.1002/14651858.CD006412.pub2, 2-s2.0-55049141508. PMC11186705 18843715

[10] Groop J. , Ketokivi M. , Gupta M. , and Holmström J. , Improving Home Care: Knowledge Creation Through Engagement and Design, Journal of Operations Management. (2017) 53–56, no. 1, 9–22, 10.1016/j.jom.2017.11.001, 2-s2.0-85034586252.

[11] Ali H. and Li H. , Use of Notification and Communication Technology (Call Light Systems) in Nursing Homes: Observational Study, Journal of Medical Internet Research. (2020) 22, no. 3, 10.2196/16252. PMC7148550 32217497

[12] Sivakanthan S. , Blaauw E. , Greenhalgh M. , Koontz A. M. , Vegter R. , and Cooper R. A. , Person Transfer Assist Systems: a Literature Review, Disability and Rehabilitation: Assistive Technology. (2021) 16, no. 3, 270–279, 10.1080/17483107.2019.1673833. 31607186

[13] Kruse C. S. , Mileski M. , Vijaykumar A. G. , Viswanathan S. V. , Suskandla U. , and Chidambaram Y. , Impact of Electronic Health Records on Long-Term Care Facilities: Systematic Review, JMIR Medical Informatics. (2017) 5, no. 3, 10.2196/medinform.7958. PMC5640822 28963091

[14] Czaja S. J. , Long-Term Care Services and Support Systems for Older Adults: The Role of Technology, American Psychologist. (2016) 71, no. 4, 294–301, 10.1037/a0040258, 2-s2.0-84965155186. 27159436

[15] Moyle W. , The Promise of Technology in the Future of Dementia Care, Nature Reviews Neurology. (2019) 15, no. 6, 353–359, 10.1038/s41582-019-0188-y, 2-s2.0-85065707464. 31073242

[16] Cheng L. M. , Choi W. P. C. , and Wong A. Y. M. , A Novel Client Service Quality Measuring Model and an eHealthcare Mitigating Approach, International Journal of Medical Informatics. (2016) 91, e16–e31, 10.1016/j.ijmedinf.2016.03.003, 2-s2.0-84962543377. 27004942

[17] Huang K. , Jiao Z. , Cai Y. , and Zhong Z. , Artificial Intelligence‐Based Intelligent Surveillance for Reducing Nurses’ Working Hours in Nurse–Patient Interaction: A Two‐Wave Study, Journal of Nursing Management. (2022) 30, no. 8, 3817–3826, 10.1111/jonm.13787. 36057432

[18] Kato K. , Yoshimi T. , Aimoto K. , Sato K. , Itoh N. , and Kondo I. , Reduction of Multiple-Caregiver Assistance Through the Long-Term Use of a Transfer Support Robot in a Nursing Facility, Assistive Technology. (2023) 35, no. 3, 271–278, 10.1080/10400435.2022.2039324. 35320681

[19] Eggleston K. , Lee Y. S. , and Iizuka T. , Robots and Labor in the Service Sector: Evidence From Nursing Homes (Working Paper No. 28322; Working Paper), 2021, National Bureau of Economic Research, https://www.nber.org/system/files/working_papers/w28322/w28322.pdf.

[20] Lu S. F. , Rui H. , and Seidmann A. , Does Technology Substitute for Nurses? Staffing Decisions in Nursing Homes, Management Science. (2018) 64, no. 4, 1842–1859, 10.1287/mnsc.2016.2695, 2-s2.0-85045206310.

[21] Qian S. , Yu P. , and Hailey D. M. , The Impact of Electronic Medication Administration Records in a Residential Aged Care Home, International Journal of Medical Informatics. (2015) 84, no. 11, 966–973, 10.1016/j.ijmedinf.2015.08.002, 2-s2.0-84942550288. 26358850

[22] Bail K. , Merrick E. , Gibson D. et al., Evaluation of ACE in Residential Aged Care: The Impact of a Digital Point-of-Care System on Residents and Staff, 2021, University of Canberra, https://www.canberra.edu.au/content/dam/uc/documents/research/arg/evaluation-of-ace-in-aged-care-cover-2F52.pdf.

[23] Koivisto T. A. , Koskela I. , Saari E. , and Ruusuvuori J. , Digitaalinen Toiminnanohjausjärjestelmä–Tukea Vai Rajoitteita Vanhushoivatyölle?, Gerontologia. (2022) 10.23989/gerontologia.113900.

[24] Leung P. P. L. , Wu C. H. , Kwong C. K. , Ip W. H. , and Ching W. K. , Digitalisation for Optimising Nursing Staff Demand Modelling and Scheduling in Nursing Homes, Technological Forecasting and Social Change. (2021) 164, 10.1016/j.techfore.2020.120512.

[25] Alastalo H. , Forsius P. , Josefsson K. et al., Teknologioiden Käytön Huomioiminen Ympärivuorokautisen Hoidon Henkilöstömitoituksessa: Arviointitutkimuksen Loppuraportti, THL Työpaperi, Finnish Institute for Health and Welfare. (2025) 2025, no. 10, Helsinki, https://urn.fi/URN:ISBN:978-952-408-474-1.

[26] Isaza L. and Cepa K. , Automation and Augmentation: A Process Study of How Robotization Shapes Tasks of Operational Employees, European Management Journal. (2024) 44, no. 1, 99–112, 10.1016/j.emj.2024.11.010.

[27] Raisch S. and Krakowski S. , Artificial Intelligence and Management: the Automation–Augmentation Paradox, Academy of Management Review. (2021) 46, no. 1, 192–210, 10.5465/amr.2018.0072.

[28] Hänninen K. , Ahtiainen H. K. , Suvikas-Peltonen E. M. , and Tötterman A. M. , Automated Unit Dose Dispensing Systems Producing Individually Packaged and Labelled Drugs for Inpatients: A Systematic Review, European Journal of Hospital Pharmacy. (2023) 30, no. 3, 127–135, 10.1136/ejhpharm-2021-003002. 34795001 PMC10176995

[29] Sinnemäki J. , Sihvo S. , Isojärvi J. , Blom M. , Airaksinen M. , and Mäntylä A. , Automated Dose Dispensing Service for Primary Healthcare Patients: A Systematic Review, Systematic Reviews. (2013) 2, no. 1, 10.1186/2046-4053-2-1, 2-s2.0-84882737420. PMC3598731 23295105

[30] Ahtiainen H. K. , Kallio M. M. , Airaksinen M. , and Holmström A.-R. , Safety, Time and Cost Evaluation of Automated and Semi-Automated Drug Distribution Systems in Hospitals: A Systematic Review, European Journal of Hospital Pharmacy. (2020) 27, no. 5, 253–262, 10.1136/ejhpharm-2018-001791, 2-s2.0-85063147251. 32839256 PMC7447254

[31] Roman C. , Poole S. , Walker C. , Smit D. V. , and Dooley M. J. , A ‘Time and Motion’ Evaluation of Automated Dispensing Machines in the Emergency Department, Australasian Emergency Nursing Journal. (2016) 19, no. 2, 112–117, 10.1016/j.aenj.2016.01.004, 2-s2.0-84960517206. 26987705

[32] Kerr D. , Wilkinson H. , and Cunningham C. , Supporting Older People in Care Homes at Night, 2008, Joseph Rowntree Foundation.

[33] Khosravi P. and Ghapanchi A. H. , Investigating the Effectiveness of Technologies Applied to Assist Seniors: A Systematic Literature Review, International Journal of Medical Informatics. (2016) 85, no. 1, 17–26, 10.1016/j.ijmedinf.2015.05.014, 2-s2.0-84937468450. 26216463

[34] Gattinger H. , Hantikainen V. , Ott S. , and Stark M. , Effectiveness of a Mobility Monitoring System Included in the Nursing Care Process in Order to Enhance the Sleep Quality of Nursing Home Residents With Cognitive Impairment, Health Technology. (2017) 7, no. 2–3, 161–171, 10.1007/s12553-016-0168-9, 2-s2.0-85034753808.

[35] Eyers I. , Carey-Smith B. , Evans N. , and Orpwood R. , Safe and Sound? Night-Time Checking in Care Homes, British Journal of Nursing. (2013) 22, no. 14, 827–830, 10.12968/bjon.2013.22.14.827, 2-s2.0-84881282584. 24260993

[36] Cahill S. , Macijauskiene J. , Nygård A.-M. , Faulkner J.-P. , and Hagen I. , Technology in Dementia Care, Technology and Disability. (2007) 19, no. 2–3, 55–60, 10.3233/TAD-2007-192-302.

[37] Creswell J. W. , Research Desing: Qualitative, Quantitative and Mixed Methods Approaches, 2014, 54, Sage Publications.

[38] Lee S.-Y. D. , Iott B. , Banaszak-Holl J. et al., Application of Mixed Methods in Health Services Management Research: A Systematic Review, Medical Care Research and Review. (2022) 79, no. 3, 331–344, 10.1177/10775587211030393. 34253078

[39] Finnish Institute for Health and Welfare , Sotkanet.fi, 2024, https://sotkanet.fi/sotkanet/fi/taulukko/?indicator=s3avtLYosta1KLbWTQIA%26region=s07MtDZxBwA=%26year=sy5zszbW0zUEAA==%26gender=t%26abs=f%26color=f%26buildVersion=3.1.5%26buildTimestamp=202505220800.

[40] Ministry of Social Affairs and Health , Government Proposal STM/2025/95, 2025, https://stm.fi/ajankohtaista/paatos?decisionId=4493.

[41] Pesonen T. , Väisänen V. , Aaltonen M. et al., Determinants of Received Care Time Among Finnish Home Care Clients and Assisted Living Facility Residents: A Time-Motion Study, BMC Geriatrics. (2024) 24, no. 1, 10.1186/s12877-024-05355-w. PMC11391809 39266978

[42] Väisänen V. , Pesonen T. , Corneliusson L. , Ruotsalainen S. , Sinervo T. , and Noro A. , Hoitoajan Jakautuminen Ikäihmisten Palveluissa Ja Päivitetty RUG-luokitus: Aikamittaus-Hankkeen Loppuraportti [Division of Care Time in Care Services for Older People and the Updated RUG-Classification: Report of the Time Measurement-Study], THL Raportti, Finnish Institute for Health and Welfare. (2022) 2022, no. 12, Helsinki, https://urn.fi/URN:ISBN:978-952-343-970-2.

[43] Colin Cameron A. and Miller D. L. , A Practitioner’s Guide to Cluster-Robust Inference, Journal of Human Resources. (2015) 50, no. 2, 317–372, 10.3368/jhr.50.2.317, 2-s2.0-84929658686.

[44] R Core Team , A Language and Environment for Statistical Computing, 2020, R Foundation for Statistical Computing, https://www.R-project.org/.

[45] Riger S. and Sigurvinsdottir R. , Thematic Analysis, Handbook of Methodological Approaches to Community-Based Research: Qualitative, Quantitative, and Mixed Methods. (2016) 33–41.

[46] Fetters M. D. , Curry L. A. , and Creswell J. W. , Achieving Integration in Mixed Methods Designs—Principles and Practices, Health Services Research. (2013) 48, no. 6pt2, 2134–2156, 10.1111/1475-6773.12117, 2-s2.0-84892970287. 24279835 PMC4097839

[47] Brown J. , Pope N. , Bosco A. M. , Mason J. , and Morgan A. , Issues Affecting Nurses’ Capability to Use Digital Technology at Work: An Integrative Review, Journal of Clinical Nursing. (2020) 29, no. 15–16, 2801–2819, 10.1111/jocn.15321. 32416029

[48] Keyworth C. , Hart J. , Armitage C. J. , and Tully M. P. , What Maximizes the Effectiveness and Implementation of Technology-based Interventions to Support Healthcare Professional Practice? A Systematic Literature Review, BMC Medical Informatics and Decision Making. (2018) 18, no. 1, 10.1186/s12911-018-0661-3, 2-s2.0-85056386281. PMC6223001 30404638

[49] Hirdes J. P. , Frijters D. H. , and Teare G. F. , The MDS‐CHESS Scale: A New Measure to Predict Mortality in Institutionalized Older People, Journal of the American Geriatrics Society. (2003) 51, no. 1, 96–100, 10.1034/j.1601-5215.2002.51017.x, 2-s2.0-0037241774. 12534853

[50] Johansson‐Pajala R. , Martin L. , Fastbom J. , and Jorsäter Blomgren K. , Nurses’ Self‐Reported Medication Competence in Relation to Their Pharmacovigilant Activities in Clinical Practice, Journal of Evaluation in Clinical Practice. (2015) 21, no. 1, 145–152, 10.1111/jep.12263, 2-s2.0-84923613157. 25327625

[51] Conte G. , Arrigoni C. , Magon A. , Stievano A. , and Caruso R. , Embracing Digital and Technological Solutions in Nursing: A Scoping Review and Conceptual Framework, International Journal of Medical Informatics. (2023) 177, 10.1016/j.ijmedinf.2023.105148. 37453178

[52] Niemeijer A. R. , Frederiks B. J. M. , Riphagen I. I. , Legemaate J. , Eefsting J. A. , and Hertogh C. M. P. M. , Ethical and Practical Concerns of Surveillance Technologies in Residential Care for People With Dementia or Intellectual Disabilities: An Overview of the Literature, International Psychogeriatrics. (2010) 22, no. 7, 1129–1142, 10.1017/S1041610210000037, 2-s2.0-79952119024. 20199699

[53] Hall T. , Kakuma R. , Palmer L. , Minas H. , Martins J. , and Armstrong G. , Intersectoral Collaboration for People-Centred Mental Health Care in Timor-Leste: A Mixed-Methods Study Using Qualitative and Social Network Analysis, International Journal of Mental Health Systems. (2019) 13, no. 1, 10.1186/s13033-019-0328-1. PMC6858633 31788024

[54] Katebi M. , Bahreini M. , Bagherzadeh R. , and Pouladi S. , Artificial Intelligence and Nursing Management: Opportunities, Challenges, and Ethical Considerations—A Scoping Review, Journal of Nursing Management. (2025) 2025, no. 1, 10.1155/jonm/2797535. PMC12328055 40771284

[55] Kiwanuka F. , Stevanin S. , Ahtisham Y. , Owusu B. , Nurmeksela A. , and Kvist T. , Nurse Leadership and Artificial Intelligence Integration in Nursing Workforce Management: A Scoping Review, Journal of Advanced Nursing. (2025) 10.1111/jan.70296. PMC13176701 41109945

[56] Gómez-Arribas S. B. , García-Herranz C. , and Calleja-Toledano P. Á. , The Challenge of Integrating Individual Complexity Into Care Models, Enfermería Clínica (English Edition). (2026) 36, no. 2, 10.1016/j.enfcle.2026.502473. 41802900

[57] Park C. S. , ‘More is Not Always Better’: Park’s Sweet Spot Theory‐Driven Implementation Strategy for Viable Optimal Safe Nurse Staffing Policy in Practice, International Nursing Review. (2023) 70, no. 2, 149–159, 10.1111/inr.12785. 35817044

[58] Park C. S.-Y. , Ethical Artificial Intelligence in Nursing Workforce Management and Policymaking: Bridging Philosophy and Practice, Journal of Nursing Management. (2025) 2025, no. 1, 10.1155/jonm/7954013. PMC11999746 40236787

