# Supplementary material for: Can Technology Use Alleviate Workforce Needs in Finnish Assisted Living Services? A Convergent Mixed‐Methods Study of Automatic Medicine Dispensers and Night‐Time Monitoring
Source: J Nurs Manag. 2026 May 8;2026:2309539. doi: 10.1155/jonm/2309539 (PMC13156071; doi:10.1155/jonm/2309539)
Supplement: Supplementary file 1 — Supporting Information 1 Time measurement form used (translated to English). [file JONM-2026-2309539-s001.pdf]

**Day of week:** Mon ☐ Tues ☐ Wed ☐ Thurs ☐ Fri ☐ Sat ☐ Sun ☐ Mon ☐

**Occupation:** Practical nurse ☐ Registered nurse ☐ Nurse assistant ☐ Other ☐

| Time | **Direct care, individual** | **Direct care, group** | **Indirect care, client not present** | **Administrative work** | **Pharmaco-therapy** | **Support tasks** | **Breaks or other** |
|---|---|---|---|---|---|---|---|
| | Only one client present | Multiple clients present | E.g., documentation, consultations | E.g., meetings, work in office | Medicine related work without client | Food, laundry, cleaning etc. | Without client |
| 6:00 | | | | | | | |
| 6:15 | | | | | | | |
| 6:30 | | | | | | | |
| 6:45 | | | | | | | |
| 7:00 | | | | | | | |
| 7:15 | | | | | | | |
| 7:30 | | | | | | | |
| 7:45 | | | | | | | |
| 8:00 | | | | | | | |
| 8:15 | | | | | | | |
| 8:30 | | | | | | | |
| 8:45 | | | | | | | |
| 9:00 | | | | | | | |
| 9:15 | | | | | | | |
| 9:30 | | | | | | | |
| 9:45 | | | | | | | |
| 10:00 | | | | | | | |
| 10:15 | | | | | | | |
| 10:30 | | | | | | | |
| 10:45 | | | | | | | |
| 11:00 | | | | | | | |
| 11:15 | | | | | | | |
| 11:30 | | | | | | | |
| 11:45 | | | | | | | |
| 12:00 | | | | | | | |
| 12:15 | | | | | | | |
| 12:30 | | | | | | | |
| 12:45 | | | | | | | |
| 13:00 | | | | | | | |
| 13:15 | | | | | | | |
| 13:30 | | | | | | | |
| 13:45 | | | | | | | |
| 14:00 | | | | | | | |
| 14:15 | | | | | | | |
| 14:30 | | | | | | | |
| 14:45 | | | | | | | |
| 15:00 | | | | | | | |
| 15:15 | | | | | | | |
| 15:30 | | | | | | | |
| 15:45 | | | | | | | |
| 16:00 | | | | | | | |
| 16:15 | | | | | | | |
| 16:30 | | | | | | | |
| 16:45 | | | | | | | |
| 17:00 | | | | | | | |
| 17:15 | | | | | | | |
| 17:30 | | | | | | | |
| 17:45 | | | | | | | |
| 18:00 | | | | | | | |
| 18:15 | | | | | | | |
| 18:30 | | | | | | | |
| 18:45 | | | | | | | |
| 19:00 | | | | | | | |
| 19:15 | | | | | | | |
| 19:30 | | | | | | | |
| 19:45 | | | | | | | |
| 20:00 | | | | | | | |
| 20:15 | | | | | | | |
| 20:30 | | | | | | | |
| 20:45 | | | | | | | |
