# Supplementary material for: Can Technology Use Alleviate Workforce Needs in Finnish Assisted Living Services? A Convergent Mixed‐Methods Study of Automatic Medicine Dispensers and Night‐Time Monitoring
Source: J Nurs Manag. 2026 May 8;2026:2309539. doi: 10.1155/jonm/2309539 (PMC13156071; doi:10.1155/jonm/2309539)
Supplement: Supplementary file 2 — Supporting Information 2 Full linear regression results. [file JONM-2026-2309539-s002.docx]

**Supplementary file 2**

**Table 1:** Full linear regression results on worktime spent during a work shift on direct care time (model 1) and medicine management and administration (model 2). Only shifts with medicine management time were included (n = 1318).

|  | **Model 1:  Direct care time (minutes)** | **Model 2:** **Medicine management and administration (minutes)** |
| --- | --- | --- |
|  | β (95 % CI) | β (95 % CI) |
| **Medicine dispensing technology in use** |  |  |
| Yes | 4.79 (-16.02 – 25.60) | **-10.45 (-16.58 – -4.32)***** |
| No (ref.) |  |  |
| **Work shift** |  |  |
| Morning (ref.) |  |  |
| Day / evening | 5.73 (-8.19 – 19.65) | -3.95 (-8.75 – 0.85) |
| Night | -15.29 (-37.46 – 6.88) | 1.05 (-10.32 – 12.42) |
| **Day of the week** |  |  |
| Monday to Friday (ref.) |  |  |
| Saturday | 9.79 (-2.33 – 21.90) | -2.72 (-7.58 – 2.14) |
| Sunday | 8.36 (-3.84 – 20.56) | 4.24 (-2.88 – 11.35) |
| **Occupation** |  |  |
| Licensed practical nurse (ref.) |  |  |
| Registered nurse | **-43.12 (-63.92 – -22.32)***** | **21.23 (12.38 – 30.08)***** |
| Nurse assistant | -29.51 (-62.22 – 3.19) | -0.33 (-13.20 – 12.55) |
| Other | **-35.79 (-70.08 – -1.50)*** | 20.63 (-19.38 – 60.65) |
| **Care organization** |  |  |
| Public (ref.) |  |  |
| Private | 3.28 (-19.54 – 26.09) | -3.60 (-9.41 – 2.22) |
| **Unit characteristics** |  |  |
| Number of employees | -0.53 (-1.76 – 0.71) | 0.05 (-0.21 – 0.32) |
| Staffing ratio (1% change) | -0.10 (-2.04 – 1.84) | 0.00 (-0.72 – 0.72) |
| Case-mix index (1% change) | **2.64 (0.08 – 5.19)*** | -0.37 (-1.00 – 0.26) |
| Adjusted R^2^ | 0.043 | 0.054 |
| *** = p < 0.001, ** = p < 0.01, * = p < 0.05  Notes: The models were adjusted for clustered standard errors on the level of the organization (n = 55). The occupation group “other” included for example therapists, physicians, and social workers. | | |

**Table 2:** Full linear regression results on worktime spent during a night shift on direct care time (model 3) and the number of care personnel per resident during a night shift (model 4). Only night shifts were included (n = 395) in model 3. Model 4 was analyzed at the organizational level (n = 52).

|  | **Model 3:**  **Direct individual care time (minutes)** | **Model 4:** **Number of care personnel per resident during night shifts** |
| --- | --- | --- |
|  | β (95 % CI) | β (95 % CI) |
| **Night-time monitoring technology in use** |  |  |
| Yes | **45.38 (9.75 – 81.00)*** | **-0.02* (-0.04** – **-0.00)** |
| No (ref.) |  |  |
| **Day of the week** |  |  |
| Monday to Friday (ref.) |  |  |
| Saturday | -8.50 (-35.38 – 18.37) |  |
| Sunday | -22.63 (-46.88 – 1.62) |  |
| **Occupation** |  |  |
| Licensed practical nurse (ref.) |  |  |
| Registered nurse | 8.24 (-56.62 – 73.10) |  |
| Nurse assistant | 56.55 (-118.73 – 231.84) |  |
| Other | -56.52 (-117.36 – 4.32) |  |
| **Care organization** |  |  |
| Public (ref.) |  |  |
| Private | -12.72 (-49.84 – 24.40) | 0.02 (-0.00 – 0.04) |
| **Unit characteristics** |  |  |
| Number of employees | 1.37 (-0.53 – 3.28) | -0.00 (-0.00 – 0.00) |
| Staffing ratio (1% change) | 3.17 (-0.33 – 6.66) | 0.00 (-0.00 – 0.00) |
| Case-mix index (1% change) | 3.36 (-0.15 – 6.87) | 0.00 (-0.00 – 0.00) |
| Adjusted R^2^ | 0.105 | 0.124 |
| *** = p < 0.001, ** = p < 0.01, * = p < 0.05  Notes: The model 3 was adjusted for clustered standard errors on the level of the organization (n = 52). The occupation group “other” included for example therapists, physicians, and social workers. | | |
